# P33 Improving compliance with early IV-to-oral switch of antibiotics using quality improvement methodology

**DOI:** 10.1093/jacamr/dlae136.037

**Published:** 2024-08-23

**Authors:** Muhammad Sami Khan, Sarah May

**Affiliations:** Respiratory Department, Calderdale Royal Hospital, Halifax, UK; Pharmacy Department, Calderdale Royal Hospital, Halifax, UK

## Abstract

**Background:**

Antimicrobial stewardship (AMS) refers to a coherent set of interventions which promote responsible use of antimicrobials.^1^ Timely IV-to-oral switch (IVOS) of antibiotics is one of the interventions which has several clinical benefits,^2^ but unfortunately frequent non-compliance has been observed in clinical practice. To address this, we aim to improve IVOS of antibiotics in the respiratory department of our hospital using quality improvement (QI) methodology.

**Methods:**

A QI project was conducted at the respiratory department of Calderdale Royal Hospital, West Yorkshire. A total of 82 adult inpatients (42 retrospective, 40 prospective), aged 18 years and over, with active prescriptions of IV antibiotics at the time of project, were elected. We excluded patients with known deep-seated infections, in the ICU or high dependency unit and those treated with IV antifungals or antivirals. UKHSA National IVOS criteria for adults^3^ was utilized as standard of care. Baseline data were collected retrospectively in November–December 2023 which was compared with post-intervention prospective re-audit in February–March 2024. Interventions included: e-mail correspondence and IVOS decision aid poster exhibition in wards; education sessions in weekly departmental teaching and board rounds; distributing lanyard cards with ACED tool, consistent with National IVOS criteria3, among doctors; and twice weekly pharmacist IVOS rounds. Outcome measures consisted of antibiotic(s) review within 24 h of prescription; eligible patients switched when met criteria; time to switch; and duration of IV therapy.

**Results:**

Post-intervention phase revealed substantial improvement in outcome measures compared with baseline: antibiotic(s) review within 24 h of prescription (from 10% to 94%); eligible patients switched when met criteria (from 33.3% to 90%); median time to switch (from 22 h 34 min to 06 h 22 min); and median duration of IV therapy (from 96 h to 48 h). We found unawareness of existence of national criteria as significant factor contributing to local non-compliance at baseline which improved with QI interventions.

**Conclusions:**

QI methodology helped significantly in promoting local antimicrobial stewardship by bridging the knowledge gaps regarding IVOS standards among doctors and empowering them for prompt IVOS decisions, where appropriate. This small AMS intervention, when implemented appropriately, has a big impact in reducing antimicrobial resistance and healthcare costs with better clinical outcomes. We recommend encouragement of such QI projects to implement standards and optimize the antimicrobial prescribing practice.
